# Case report: Multidrug-resistant *Streptococcus pseudoporcinus* isolated from an infected surgical wound of a 9-year-old spayed female Great Dane dog

**DOI:** 10.3389/fvets.2023.1139381

**Published:** 2023-03-03

**Authors:** Yesutor K. Soku, Athema L. Etzioni, Abdelrahman Mohamed

**Affiliations:** ^1^Environmental and Aquatic Research Laboratory, Department of Pathobiology, College of Veterinary Medicine, Tuskegee University, Tuskegee, AL, United States; ^2^Microbiology Diagnostic Laboratory, Department of Pathobiology, College of Veterinary Medicine, Tuskegee University, Tuskegee, AL, United States; ^3^Clinical Pathology Diagnostic Laboratory, Department of Pathobiology, College of Veterinary Medicine, Tuskegee University, Tuskegee, AL, United States

**Keywords:** drug resistance, bacteria, infection, microbiology, case report, purulent discharge

## Abstract

*Streptococcus pseudoporcinus* (*S. pseudoporcinus*) is a β-hemolytic, Gram-positive novel bacterium first identified in 2006. It is a catalase-negative, non-motile coccus arranged in short chains. Furthermore, it has a broad beta-hemolytic reaction on sheep blood agar and cross-reacts with Lancefield group B antigen agglutination reagent. In this study, we report a case of *S. pseudoporcinus* infection of a surgical wound on the left metatarsus of a dog. The patient is a 9-year-old spayed female Great Dane dog with a brief history of multiple cutaneous masses being removed. Post-surgery, the post-surgical site on the left metatarsus became infected and discharged purulent material with a fetid odor. Upon preliminary diagnostic testing, we detected catalase-negative Gram-positive cocci exhibiting beta-hemolytic growth on sheep blood agar. A VITEK^®^ 2 Compact machine from bioMérieux identified the bacterium as *S. pseudoporcinus*. Furthermore, antibiotic testing revealed multidrug resistance. Therefore, we document a multidrug-resistant *S. pseudoporcinus* isolate as a cause of canine post-surgical wound infection. Furthermore, it was the only isolate detected from the sample; hence, it is the cause of the infection. To our knowledge, this case is the first report of *S. pseudoporcinus* in a dog.

## 1. Introduction

*Streptococcus pseudoporcinus* was first described in 2006 after isolates were recovered from the genitourinary tract of women (1). Although phenotypically identical to *Streptococcus porcinus*, 16S ribosomal ribonucleic acid (16S rRNA) gene sequencing differentiated these two bacteria (1, 2). On blood agar plates, colonies were small, round, and circular with β-hemolysis. This characteristic is similar to biochemical profiles shown by *Streptococcus agalactiae* (group B *Streptococcus*, GBS) (3). Consequently, this cross-reaction with standard GBS test kits raises concerns about the misdiagnosis of this bacterium in suspected GBS cultures. The Centers for Disease Control and Prevention (CDC) has added this bacterium to their *Streptococcus* Laboratory collections, making it easier for diagnosticians to diagnose it from GBS (4). In addition, this bacterium has often been recovered from pregnant women, infants, and other immunocompromised patients (5–7). While re-evaluating 97 *S. porcinus* isolates from animal, human, and dairy sources in the CDC *Streptococcus* strain collection using 16S rRNA and rpoB gene sequencing, none identified as *S. pseudoporcinus* were from animals. To our knowledge, we report the first case of *S. pseudoporcinus* from an infected surgical wound in a dog.

## 2. Case description

A 9-year-old spayed female Great Dane dog was presented to the Tuskegee University Small Animal Veterinary Medical Teaching Hospital for evaluation of multiple cutaneous masses, one of which was on the left metatarsus. The histopathology result indicated a benign tumor in this location. Dexmedetomidine was used for premedication; cerenia, hydromorphone, and ketamine were also administered; and propofol was administered for induction/maintenance during the mass removal surgery. Some masses were surgically removed, including the one on the left metatarsus. The patient was discharged the same day with instructions for post-surgical care while at home, and the owner was prescribed the antibiotic enrofloxacin and anti-inflammatory carprofen for post-operative care. However, 10 days after the operation, the patient returned to the hospital with an inflamed surgical wound on the left metatarsus that oozed purulent material with a fetid odor.

A wound swab was submitted to the Microbiology Laboratory Service at Tuskegee University College of Veterinary Medicine (TUCVM) for aerobic and anaerobic culture and susceptibility testing. After 24 h of incubation, there was no growth on MacConkey agar, but there was growth on trypticase soy agar (TSA). Pinpoint, round, creamy colonies grew on the aerobic culture plate and candle jar culture plate, with the latter revealing more colony growth. Gram stain revealed short chains of Gram-positive cocci, while catalase testing showed no formation of air bubbles. The isolate was subcultured on Brain Heart Infusion blood agar (5% sheep blood) and incubated in a candle jar at 37°C overnight. After 20 h of incubation, a wide beta-hemolysis zone was observed around bacterial colonies. The isolate was re-cultured on TSA and incubated overnight at 37°C. The Gram-positive VITEK^®^ 2 microplate from bioMérieux was inoculated with fresh TSA culture and set in the machine according to the manufacturer's protocol. After 5 h, the machine identified the isolate as *S. pseudoporcinus* with excellent identification.

Antibiotic susceptibility testing was carried out using an agar disk diffusion test and a Beckman Coulter Gram-positive microplate (Brea, CA). According to Clinical and Laboratory Standards Institute (CLSI) (M100-S25) guidelines, two plates of Muller-Hinton agar were seeded from 0.5 McFarland bacterial suspension, and the antibiotic disks were placed on the plates. The plates were incubated in a candle jar at 37°C for 20 h. Similarly, following the manufacturer's instructions, the Beckman Coulter microplate was prepared using the Renok rehydration system to determine the drug breakpoint concentrations using CLSI guidelines for Gram-positive bacteria. The rehydrated plate was incubated in a candle jar at 37°C for 20 h. The results of the diameter for the zone of inhibition following agar disk diffusion are shown in Table 1. Similarly, the interpretive results of the isolates tested against various concentrations of antibiotics using the Beckman Coulter microplate are shown in Table 2.

**Table 1:** Agar diffusion test results.

**Antimicrobial classes**	**Antimicrobial agents**	**Inhibition zone ranges** ^**a**^		**Plate 1**	**Plate 2**	**Average**	**Interpretation**
		**Resistance**	**Susceptible**				
Potentiated sulfonamides	Trimethoprim-sulfamethoxazole	≤ 15	≥19	33	35	34.5	S
Potentiated penicillins	Amoxicillin–clavulanic acid	≤ 19	≥20	0	0	0	R
Penicillins	Ampicillin^b^	–	≥24	0	10	5	R
Peptides	Vancomycin^b^	–	≥17	0	0	0	R
Carbapenems	Meropenem	≤ 13	≥16	22	23	22.5	S
Cephalosporins	Cefoxitin	≤ 14	≥18	0	0	0	R
Macrolides	Azithromycin	≤ 13	≥18	19	18	18.5	S
Aminoglycosides	Gentamycin	≤ 12	≥15	23	24	23.5	S
	Tobramycin	≤ 12	≥15	26	24	25	S
Tetracyclines	Doxycycline	≤ 12	≥16	27	26	26.5	S
Lincosamides	Clindamycin	≤ 15	≥19	0	0	0	R
Phenicols	Chloramphenicol	≤ 17	≥21	20	20	20	I
Ansamycins	Rifampin	≤ 16	≥19	0	0	0	R
Fluoroquinolones	Ciprofloxacin	≤ 15	≥19	32	31	31.5	S

^a^Inhibition zone diameter in mm for Streptococci quoted from Sensi-Disk BD (R, resistant; S, sensitive; I, intermediate resistant).

^b^These drugs do not have an intermediate resistant range, resulting in either S or R.

**Table 2:** Beckman Coulter microplate breakpoint results.

**Antimicrobial classes**	**Antimicrobial agents**	**Growth**^**a**^ **at BP concentrations**^**b**^												**Result**
		**0**	**0.03**	**0.12**	**0.25**	**0.5**	**1**	**2**	**4/(4/4)**	**8/(8/4)**	**16/(16/8)**	**32**	**64**	
Potentiated penicillins	Ampicillin-sulbactam										16/8			R
	Piperacillin-Tazobactam	0												S
Penicillins	Penicillin									8				R
	Ampicillin									8				R
	Oxacillin							2						R
Cephalosporins	Cefazolin										16			R
	Ceftriaxone	0												S
	Ceftaroline					0.5								S
Carbapenems	Meropenem	0												S
Peptides	Vancomycin										16			R
	Daptomycin								4					R
Lincosamides	Clindamycin								4					R
Aminoglycosides	Gentamycin	0												S
Macrolides	Azithromycin								4					R
Tetracyclines	Tetracycline	0												S
Ansamycins	Rifampin							2						R
Oxazolidinones	Linezolid								4					R
Macrolides	Erythromycin								4					R
Fluoroquinolones	Ciprofloxacin	0												S
	Levofloxacin	0												S
Steroidal antibiotic	Fusidic acid	0												S

^a^Bacterial growth at higher concentrations was observed in μg.

^b^Breakpoint values for the tested drug concentration in μg.

## 3. Discussion

We report, to our knowledge, a novel case of *S. pseudoporcinus* in a dog that presented oozing purulent material with a fetid odor 10 days post-surgery. Microbiological testing on the wound swab revealed the causative agent as *S. pseudoporcinus*. This bacterium is a usual colonizer of healthy female genitourinary tracts and has been implicated in liver cirrhosis, leg cellulitis, endocarditis, thumb infection, and fetal demise (3, 5–9) and has also been associated with bacteremia in a patient co-infected with Syphilis–HIV (5, 10). In the present study, we present for the first time the isolation of *S. pseudoporcinus* from an infected wound in a dog. With *S. pseudoporcinus* described as a human strain of *Streptococcus* spp. and differentiated from *S. porcinus* by 16S rRNA (1), this case may be considered a reverse zoonosis even though the mechanism of infection in this dog is unclear.

Multidrug resistance (MDR) is non-susceptibility to at least one agent in three or more antimicrobial classes (11). Based on this definition, this study defined MDR based on disk diffusion assay and microplating results; *S. pseudoporcinus* exhibited resistance to six out of 14 antimicrobial agents and 12 out of 21 antimicrobial agents after agar disk diffusion and Beckman Coulter microplating, respectively. *Streptococcus pseudoporcinus* showed resistance to ansamycins, peptides, lincosamides, penicillins, and first- and second-generation cephalosporins. On the contrary, *S. pseudoporcinus* was susceptible to carbapenems, aminoglycosides, fluoroquinolones, phenicols, tetracyclines, third- and fifth-generation cephalosporins, potentiated sulfonamides, and fusidic acid. Our finding is similar to studies reporting the susceptibility of *S. pseudoporcinus* to fluoroquinolones, tetracyclines (8), and Trimethoprim–sulfamethoxazole (12). Macrolides (including erythromycin and azithromycin) were resistant based on the breakpoint results from microplating but differed in the agar disk diffusion results. Determining the antimicrobial resistance genes present was not achieved in this study as this study focused on the diagnostic and antibiotic susceptibility profiles of the bacterial isolate confirmed to suggest the best treatment course for the dog. Therefore, since this phenotypic-based MDR was observed in *S. pseudoporcinus* recovered from a dog for the first time in the literature, this study reports this as a novel case of an MDR *S. pseudoporcinus* in a dog.

In conclusion, we described the first report of *S. pseudoporcinus* in a dog. Because *S. pseudoporcinus* is an emerging multidrug-resistant pathogen in humans, its isolation in dogs is a worrying concern, as this could lead to a potential public health menace.

## Data availability statement

The original contributions presented in the study are included in the article/supplementary material, further inquiries can be directed to the corresponding author.

## Author contributions

YS wrote the second and preceding drafts of the manuscript and contributed to the conception and design of the study. AE organized and included the database from the clinic. AM diagnosed the case, contributed to the conception and design of the study, and wrote the first draft of the manuscript. All authors contributed to the manuscript revision and read and approved the submitted version.
